# Response to Bile Salts in Clinical Strains of *Acinetobacter baumannii* Lacking the AdeABC Efflux Pump: Virulence Associated with Quorum Sensing

**DOI:** 10.3389/fcimb.2017.00143

**Published:** 2017-05-09

**Authors:** Maria López, Lucia Blasco, Eva Gato, Astrid Perez, Laura Fernández-Garcia, Luis Martínez-Martinez, Felipe Fernández-Cuenca, Jesús Rodríguez-Baño, Alvaro Pascual, German Bou, Maria Tomás

**Affiliations:** ^1^Department of Microbiology, Complejo Hospitalario Universitario A Coruña-INIBICLa Coruña, Spain; ^2^Spanish Network for Research in Infectious Diseases (REIPI RD12/0015), Hospital Virgen MacarenaSeville, Spain; ^3^Department of Clinical Microbiology, Hospital Universitario Marqués de Valdecilla-IFIMAVSantander, Spain; ^4^Departament of Molecular Biology, University of CantabriaSantander, Spain; ^5^Department of Microbiology and Infectious Diseases, Hospital Universitario Virgen MacarenaSeville, Spain; ^6^Department of Medicine, Universidad de SevilleSeville, Spain

**Keywords:** *Acinetobacter baumannii*, bile salts, quorum sensing, type VI secretion

## Abstract

**Introduction:** *Acinetobacter baumannii* is an opportunistic nosocomial pathogen associated with multiple infections. This pathogen usually colonizes (first stage of microbial infection) host tissues that are in contact with the external environment. As one of the sites of entry in human hosts is the gastrointestinal tract, the pathogen must be capable of tolerating bile salts. However, studies analyzing the molecular characteristics involved in the response to bile salts in clinical strains of *A. baumannii* are scarce.

**Material and Methods:** Microbiological and transcriptional studies (arrays and RT-PCR) in the response to bile salts were carried out in isogenic (*A. baumanni* Δ*adeB* ATCC 17978 and *A. baumannii* Δ*adeL* ATCC 17978) and clinical strains from clone ST79/PFGE-HUI-1 which is characterized by lacking the AdeABC efflux pump and by overexpression the AdeFGH efflux pump.

**Results and Discussion:** In presence of bile salts, in addition to the glutamate/aspartate transporter were found overexpressed in *A. baumannii* Δ*adeB* ATCC 17978, the virulence factors (surface motility, biofilm, and Type VI Secretion System) which are associated with activation of the Quorum Sensing system. Overexpression of these factors was confirmed in clinical strains of clone ST79/PFGE-HUI-1.

**Conclusions:** This the first study about the adaptive response to bile salts investigating the molecular and microbiological characteristics in response to bile salts of an isogenic model of *A. baumannii* ATCC 17978 and clinical isolates of *A. baumannii* (clinical strains of ST79/PFGE-HUI-1) lacking the main RND efflux pump (AdeABC). Clinical isolates of *A. baumannii* lacking the AdeABC efflux pump (clone ST79/PFGE-HUI-1) displayed a new clinical profile (increased invasiveness) possibly associated with the response to stress conditions (such as the presence of bile salts).

## Introduction

*Acinetobacter baumannii* is an important pathogen that is known to be a major agent in healthcare-associated and nosocomial infections (Antunes et al., 2013). The pathogen is increasingly involved in hospital outbreaks of infection, particularly in Intensive Care Units (ICUs) (del Mar Tomas et al., 2005). In most of these outbreaks, various inanimate objects in the hospital environment have been identified as the principal source of infection (Sherertz and Sullivan, 1985; Cefai et al., 1990). However, the genus *Acinetobacter* is known to be a normal inhabitant of human skin. Some researchers have therefore postulated that, in the context of an outbreak of *A. baumannii* infection, humans may be transient skin carriers, thus facilitating cross-contamination and also representing a potential source of hospital spread of the infection (Mulin et al., 1995). The digestive tract of ICU patients is an important reservoir of multiresistant *A. baumannii* in hospital settings (Corbella et al., 1996). The types of surveillance samples most frequently analyzed in patients include sputum and tracheostomy exudate, wounds, armpit/groin, and rectal smears. In a Spanish study involving the detection of *A. baumannii* in different surveillance samples from ICU patients, the microorganism was identified in 75% of axillary-pharyngeal samples and in 77% of rectal swabs; the pathogen was identified in 90% of patients following analysis of a combination of axillary/rectal/pharyngeal specimens and in 96% of patients following analysis of a combination of pharyngeal-rectal samples (Rodríguez-Baño et al., 2004).

Several factors affect the ability of the microorganism to persist in high numbers in the gut, both as a commensal and as an opportunistic pathogen. One such factor is tolerance to bile salts (Pumbwe et al., 2007). Bile salts (i.e., salts of bile acids) are formed from secondary bile acids (bile acids conjugated to amino acids) which attach to a sodium or potassium ion to form a salt. Bile acids are retained in the gallbladder as bile salts and are secreted into the intestine (Malik, 2016). Two mechanisms of tolerance to bile salts have been identified in bacteria to date: RND efflux pumps (Lin et al., 2003; Pumbwe et al., 2008) and glutamate transporters (Krastel et al., 2010).

Three types of RND efflux pumps have been described in clinical strains of *A. baumannii*: AdeABC (expression of which is controlled by AdeRS); AdeIJK (in which the regulatory gene is *adeN*); and AdeFGH (in which *adeL* is the negative regulatory gene). The AdeFGH pump and in particular the AdeABC pump play a major role in acquired resistance (Coyne et al., 2010; He et al., 2015), whereas the AdeIJK pump is responsible for intrinsic resistance (Coyne et al., 2011). Moreover, overexpression of AdeABC and AdeFGH efflux pumps has been associated with increased biofilm production (He et al., 2015; Yoon et al., 2015; Richmond et al., 2016). However, neither these RND efflux pumps nor glutamate transporters have been associated with tolerance to bile salts in *A. baumannii* strains.

Finally, several studies have investigated how the virulence factors associated with the Quorum Sensing (QS) system can be regulated differently throughout the intestine by bile salts and, therefore, by the different commensal bacteria present (Zheng et al., 2010; Bachmann et al., 2015). The QS system enables bacterial populations to live and proliferate in an environment (sometimes hostile) with effective intercellular communication. However, this has not yet been investigated in strains of *A. baumannii*.

In this study, we carried out microbiological and transcriptional studies to investigate the response to bile salts in clinical strains of *A. baumannii* (clone ST79/PFGE-HUI-1) lacking the AdeABC efflux pump, as well as in isogenic mutant strains of *A. baumannii* ATCC 17978.

## Materials and methods

### Strains, susceptibility testing, and growth with bile salts

#### Isogenic and clinical strains

*A. baumannii* ATCC 17978 was used as a reference strain. This strain was used to produce two stable mutants with the pMo130 plasmid; following the instructions of Hamad and colleagues (Hamad et al., 2009), *A. baumannii* Δ*adeB* ATCC 17978 and *A. baumannii* Δ*adeL* ATCC 17978 mutants were obtained. The mutants were confirmed by sequencing analysis and RT-PCR assays (Rumbo et al., 2013). The primers used are listed in Table S1 (Supplementary Material).

The Ab421 GEIH-2010 strain and other 10 clinical strains of *A. baumannii*, all belonging to clone ST79/PFGE-HUI-1 and identified during the second multicenter Spanish study of this pathogen (GEIH-REIPI-2010-Ab project), were included in the present study. These isolates were characterized in a previous study (Rumbo et al., 2013). Species identification was confirmed by detection of the *bla*_OXA51_ gene, and the *adeA, adeB, adeC, adeR*, and *adeS* genes were not detected (Rumbo et al., 2013).

To confirm the absence of the AdeABC system and regulatory genes, we applied Next Generation Sequencing (NGS) to a representative clinical strain (Ab421 GEIH-2010) of clone ST79/PFGE-HUI-1. The genome of this strain was recently published in the Genome Announcements (Lopez et al., 2016). Moreover, the LysR-type regulator protein (AdeL) upstream of the AdeF protein (AdeFGH) contained a new mutation that introduced an amino acid substitution (Met7 → Stop) (Lopez et al., 2016).

The antibiotic susceptibility profile (isogenic and Ab421 GEIH-2010 strain) was determined by microdilution, according to CLSI recommendations (CLSI, 2015). The MICs were determined in the presence of bile salts (cholic acid sodium salt 50% and deoxycholic acid sodium salt 50%, Sigma Aldrich, Germany) and Phe-Arg β-naphthylamide dihydrochloride (PAbetaN), a commonly assumed RND efflux pump inhibitor (Pannek et al., 2006). The bile salts were used at a concentration 0.5% as the physiological concentration in the human intestine ranges between 0.1 and 1.3% (Pumbwe et al., 2007).

#### Bacterial growth in the presence of bile salts

Clinical strains (Ab421, Ab427, Ab428, Ab435, and Ab436) of *A. baumannii* clone ST79/PFGE-HUI-1 (two biological replicates of each strain) were grown in LB at 37°C and 180 rpm. After incubation of the cultures overnight, the optical density (OD) was measured and adjusted to 0.02 OD_600_ in modified LB-LN (medium low nutrients comprising 2 g/L tryptone, 1 g/L yeast extract, and 5 g/L NaCl) supplemented with bile salts at 0.5%. Cultures were incubated at 37° (static conditions) and the growth was monitory at different times (3, 6, 9, 12, 24, 36, and 48 h) in a Zuzi 4250/20 spectrophotometer (Jin et al., 1998) until an OD_600_ 0.4 was reached. The *A. baumannii* ATCC 17978 strain was included as a control.

### Surface motility

Motility assays were performed in 6-well plates containing three types of Luria broth: (i) normal LB medium (10 g/L tryptone, 5 g/L yeast extract, and 10 g/L NaCl); (ii) modified LB-LS (medium low salts which is constituted by 10 g/L tryptone, 5 g/L yeast extract, and 5 g/L NaCl) (47); and (iii) modified LB-LN plus 0.3% Eiken agar (López et al., 2017). These media were supplemented with 0.5 and 1% bile salts, except for the controls (no supplementation).

Strains *A. baumannii* ATCC 17978 (and mutants thereof) and Ab421 GEIH-2010 were inoculated in LB broth (Normal LB, Modified LB-LS, and Modified LB-LN) and incubated overnight at 37°C. An aliquot of 1 μl of the overnight culture was spotted in the center of each well and the plates were incubated at 37°C. Migration was measured after overnight incubation of the culture. The average diameter of the zone of surface motility was determined, and the isolates were classified as non-motile (NM, <5 mm), intermediately motile (IM, 5–20 mm), and highly motile (HM, >20 mm).

### Biolfim experiments

#### Scanning electron microscopy (SEM) studies

Overnight cultures (two biological replicates of each strain) of *A. baumannii* were used to inoculate 5 mL of modified LB-LN in 50 ml conical tubes at a 1:100 dilution. The test medium was supplemented with 0.5% bile salts, and the control medium was not supplemented. Sterile polystyrene coverslips were placed in the inoculated 50 mL conical tubes, which were incubated for 48 h at 37°C without shaking, as previously described (Gaddy et al., 2009). Coverslips were washed, dehydrated in ethanol, processed with a critical point drier, and sputter-coated, as described above (Tomaras et al., 2003). Biofilms formed above, at and below the liquid-air interface were viewed in a Zeiss Supra Gemini Series 35 V scanning electron microscope, as previously described (Rey et al., 1989).

#### Quantitative assays

Biofilm formation was quantified following the procedure described by Álvarez-Fraga et al. (2016). The strains were grown on Luria Broth for 18 h at 37°C and used to inoculate 5 mL of LB broth. Cultures were grown at 37°C with shaking. Overnight cultures were pelleted, washed and resuspended in 5 mL of modified LB-LN in presence and absence of 0.5% bile salts. A 1:100 dilution of each strain was incubated at 37°C for 48 h under static conditions. Growth of the culture was measured at OD_600_ to estimate total cell biomass. Biofilm formation was quantified by staining with crystal violet and solubilized with ethanol-acetone. The OD_580_/OD_600_ ratio was used to normalize the amount of biofilm formed to the total cell content of each sample tested, to overcome variations due to differences in bacterial growth under several experimental conditions. Eight independent replicates were considered. A student's *t*-test was performed to evaluate the statistical significance of the observed differences between the strains considered.

### Gene expression

Gene expression studies were carried out by microarray and RT-PCR analysis. In both types of analysis, RNA was isolated using hot phenol extraction and subjected to DNase I treatment (Invitrogen). The RNA was then cleaned on an RNeasy column (Qiagen) following the manufacturer's mini cleanup protocol (Hamner et al., 2013) to obtain Dnase-treated RNA from late log-phase cultures in LB-LN (OD = 0.4–0.6) in the absence and presence of 0.5% bile salts in static conditions and tigecyline (0.5 mg/L). The RNA samples were quantified in a NanoDrop ND-1000 Spectrophotometer (NanoDrop Technologies). The quality and integrity of the samples were determined in an Agilent 2100 Bioanalyzer with RNA 6000 Nano reagents and RNA Nano Chips (Agilent Technologies), and only samples with an RNA integrity number (RIN) >8 were included. Analysis of controls without reverse transcriptase confirmed the absence of contaminating DNA in the samples.

The *A. baumannii* strain ATCC 17978 and mutants *A. baumannii* Δ*adeL* ATCC 17978 and *A. baumannii* Δ*adeB* ATCC 17978 were included in the microarrays (Bioarray Diagnostico Genetico, Alicante, Spain). The analysis was conducted using eArray (Agilent) (Aranda et al., 2013). Labeling was carried out by two-color microarray-based prokaryote analysis by Fair Play III labeling, version 1.3 (Agilent). Four independent RNA extractions per condition (biological replicates) were used in each experiment. Statistical analysis was carried out with the Bioconductor software package RankProd for the R computing environment. A gene was considered induced when the ratio of the treated to the untreated preparation was ≥1.5 and the *P*-value was < 0.05.

For RT-PCR assays, we used the Ab421 GEIH-2010 and other clinical isolates (Ab427, Ab428, Ab435, and Ab436) of ST79/PFGE-HUI-1 clone and *A. baumannii* Δ*adeL* ATCC 17978 strains. The following were analyzed by RT-PCR: (i) expression of the *adeG* gene (AdeFGH efflux pump) in the strains cultured in the presence of bile salts (0.5%) and tigecycline (0.5 mg/L), and (ii) expression of genes determined by microarray analysis (A1S_1490, A1S_0115, A1S_1295, and A1S_1510). The studies were carried out with the Lightcycler 480 RNA MasterHydrolysis Probe (Roche, Germany). The UPL Taqman Probes (Universal Probe Library-Roche, Germany) and primers used are listed in Table S1.

The concentrations of the samples were adjusted to efficiencies of 90–110% (50 ng of RNA), and all experiments were performed in triplicate from three RNA extractions. For each strain, the expression of all genes was normalized relative to that of the *rpoB* gene. The normalized expression of each gene of interest was then calibrated relative to its expression by strains cultured in the absence of the bile salts or tigecycline in *A. baumannii* ATCC 17978, which were assigned a value of 1.0 (Mean Relative Expression, RE) (Aranda et al., 2013). Overexpression of the gene was defined by RE values of ≥ 1.5 (Tomas et al., 2010).

## Results

### Antimicrobial susceptibility in *A. baumannii* ATCC 17978 isogenic model and Ab421 GEIH-2010 (Table 1)

Resistance of the *A. baumannii* strains to several antimicrobials increased in the presence of 0.5% bile salts, although not significantly, which may indicate that expression of the AdeFGH efflux pump (RND type) is not modulated by bile salts.

**Table 1:** **MICs of different antimicrobial agents against clinical and isogenic strains of *A. baumannii* in the presence or absence of bile salts (0.5%) or in the presence of Phe-Arg β-naphthylamide dihydrochloride (PAbetaN, 100 μg/mL)**.

**Strain**	**Antimicrobial**	**MIC (mg/L)**	**MIC (mg/L-Bile Salts 0.5%)**	**MIC (mg/L-PAßN 100 μg/mL)**
*A. baumannii* ATCC 17978	Tobramycin	0.5	0.5	0.5
	Sulfamethoxazole	4,864	4,864	4,864
	Tigecycline	1	2	2
	Norfloxacin	4	2	4
	Ciprofloxacin	0.5	0.25	0.25
	Gentamicin	1	1	1
	Tetracycline	4	1	1
	Netilmicin	1	1	1
*A. baumannii* ATCC 17978 Δ*adeB*	Tobramycin	0.25	1	0.5
	Sulfamethoxazole	4,864	4,864	4,864
	Tigecycline	1	1	1
	Norfloxacin	4	4	4
	Ciprofloxacin	0.5	0.5	0.125
	Gentamicin	0.5	2	0.5
	Tetracycline	4	4	1
	Netilmicin	1	1	0.5
*A. baumannii* ATCC 17978 Δ*adeL*	Tobramycin	1	1	0.25
	Sulfamethoxazole	4,864	4,864	4,864
	Tigecycline	1	0.5	0.25
	Norfloxacin	8	8	0.5
	Ciprofloxacin	0.5	2	0.25
	Gentamicin	1	4	0.5
	Tetracycline	4	4	1
	Netilmicin	2	1	0.5
Ab421 GEIH-2010	Tobramycin	64	64	8
	Sulfamethoxazole	9,728	9,728	4,864
	Tigecycline	16	16	4
	Norfloxacin	1,024	2,048	512
	Ciprofloxacin	512	512	512
	Gentamicin	512	512	128
	Tetracycline	16	32	8
	Netilmicin	256	256	8

The MICs of tobramycin, tigecycline, norfloxacin, ciprofloxacin, gentamicin, tetracycline, and netilmicin were lowered in *A. baumannii* strain Δ*adeL* ATCC 17978 in the presence of the RND-efflux pump inhibitor Phe-Arg β-naphthylamide dihydrochloride (PAbetaN). These results confirm the overexpression of the AdeFGH efflux pump in relation to antibiotic resistance. Moreover, the levels of expression of the *adeG* gene (AdeFGH) revealed by RT-PCR explained the results of the MIC assays. The relative expression (RE) of the *adeG* gene in *A. baumannii* Δ*adeL* ATCC 17978 strains cultured in the presence of 0.5% bile salts was 0.5 (i.e., it was not overexpressed relative to expression in the absence of bile salts). However, the level of expression of *adeG* was 2.53 times higher in the presence than in the absence of tigecycline (0.5 mg/L).

In clinical strain Ab421 GEIH-2010 (belonging to clone ST79/PFGE-HUI-1), the MICs of tobramycin, tigecycline, norfloxacin, ciprofloxacin, gentamicin, tetracycline, and netilmicin decreased (by 2–4 times) in the presence of PAbetaN. The RE was also 2.71 times higher in the presence than in the absence of tigecycline (0.5 mg/L) in this isolate.

### Virulence phenotype: surface motility and biofilm (*A. baumannii* ATCC 17978 isogenic model and Ab421 GEIH-2010)

**Figure 1:**
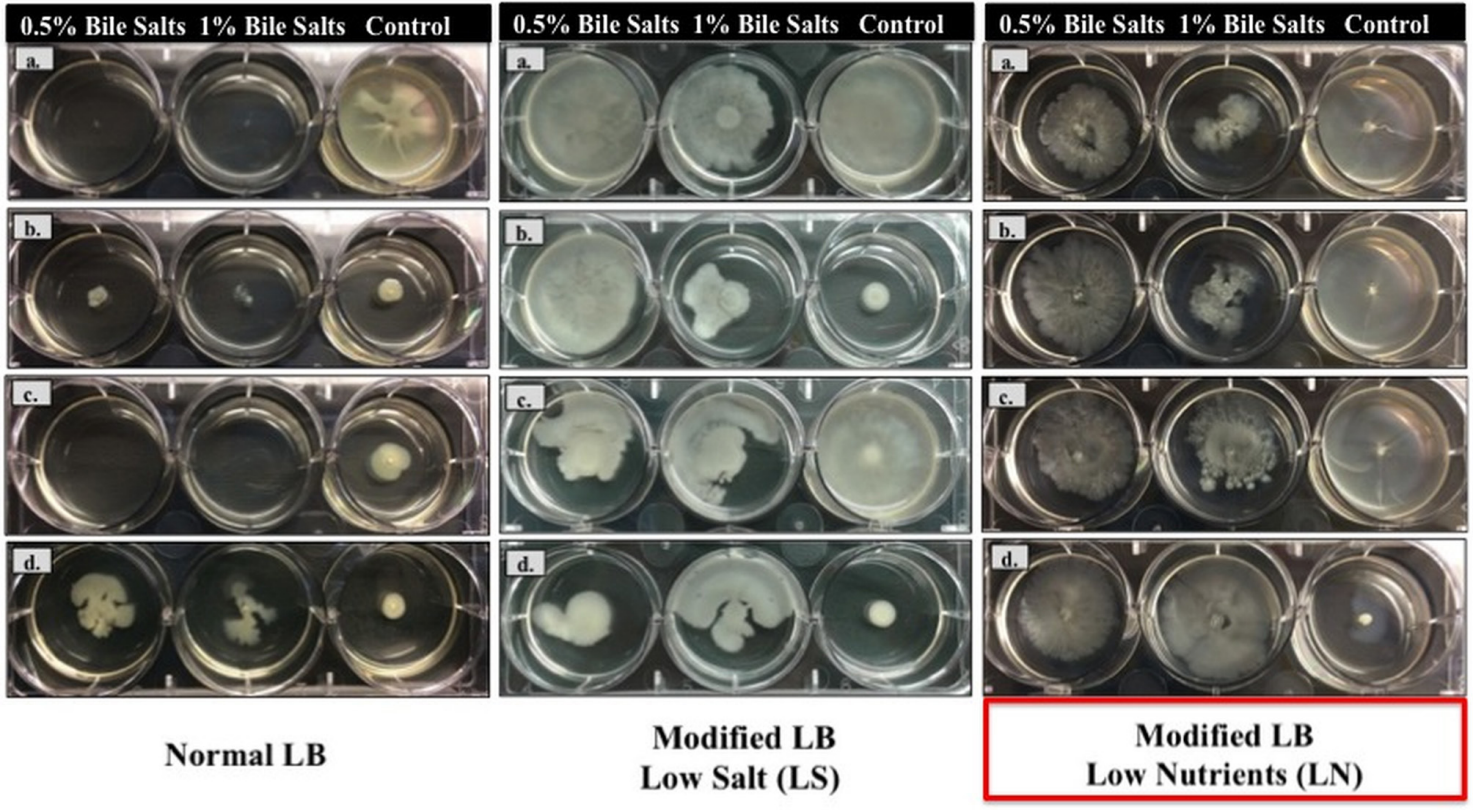
**Motility of *A. baumannii* strains in the presence of 0.5 and 1% of bile salts in three types of LB broth (Normal LB medium, Modified LB-LS and Modified LB-LN)**. **(a)** *A. baumannii* ATCC 17978; **(b)** *A. baumannii* Δ*adeB* ATCC 17978; **(c)** *A. baumannii* Δ*adeL* ATCC 17978; and **(d)** Ab421 GEIH-2010. The arrows on the left show the similarity in the behavior of both strains (*A. baumannii* Δ*adeB* ATCC 17978 and Ab421 GEIH-2010) in different medium and in presence of bile salts.

In relation to surface motility studies in Normal LB (Figure 1), the *A. baumannii* ATCC and *A. baumannii* ATCC 17978Δ*adeL* strains did not grow, while *A. baumannii* Δ*adeB* ATCC 17978 and Ab421 GEIH-2010 both grew in the presence of 0.5 and 1% bile salts. The surface motility of isogenic strains (*A. baumannii* ATCC 17978 and mutants) was also higher in the presence of bile salts (0.5%) in this modified medium-Low Salt (LB-LS). However, the greatest increase in the motility of strain Ab421 GEIH-2010 cultured in presence of bile salts (0.5%) was observed in modified LB-Low Nutrients (LB-LN). Therefore, this concentration of bile salts (0.5%) and the modified medium LB-LN were used for all further experiments in this study.

**Figure 2:**
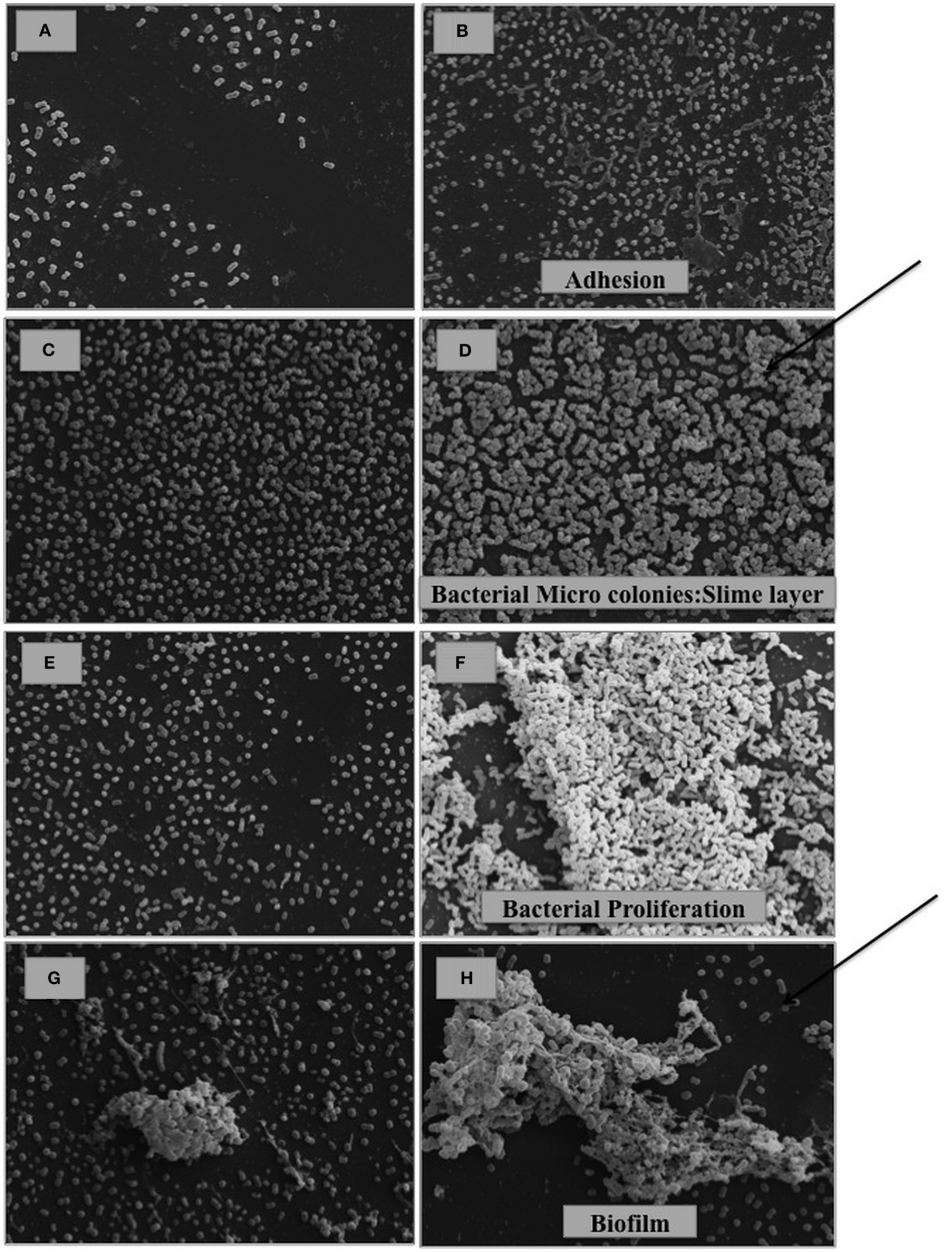
**SEM analysis of *A. baumannii* cells cultured in the absence (A,C,E,G)** and presence of 0.5% bile salts **(B,D,F,H)**. **(A,B)** *A. baumannii* ATCC 17978; **(C,D)** *A. baumannii* Δ*adeB* ATCC 17978; **(E,F)** *A. baumannii* Δ*adeL* ATCC 17978; **(G,H)** Ab421 GEIH-2010. (Scale bars: 20 μm). It is observed in presence of bile salts, the state of adhesion in *A. baumannii* ATCC 17978 **(B)**, slime layer-micro colonies (previous state of biofilm formation) in *A. baumannii* Δ*ade*B ATCC 17978 **(D)**, proliferation in *A. baumannii* Δ*ade*L ATCC 17978 **(F)** and finally, biofilm formation in Ab421 GEIH-2010 **(H)**. The arrows indicate the most advanced stages of biofilm development.

**Figure 3:**
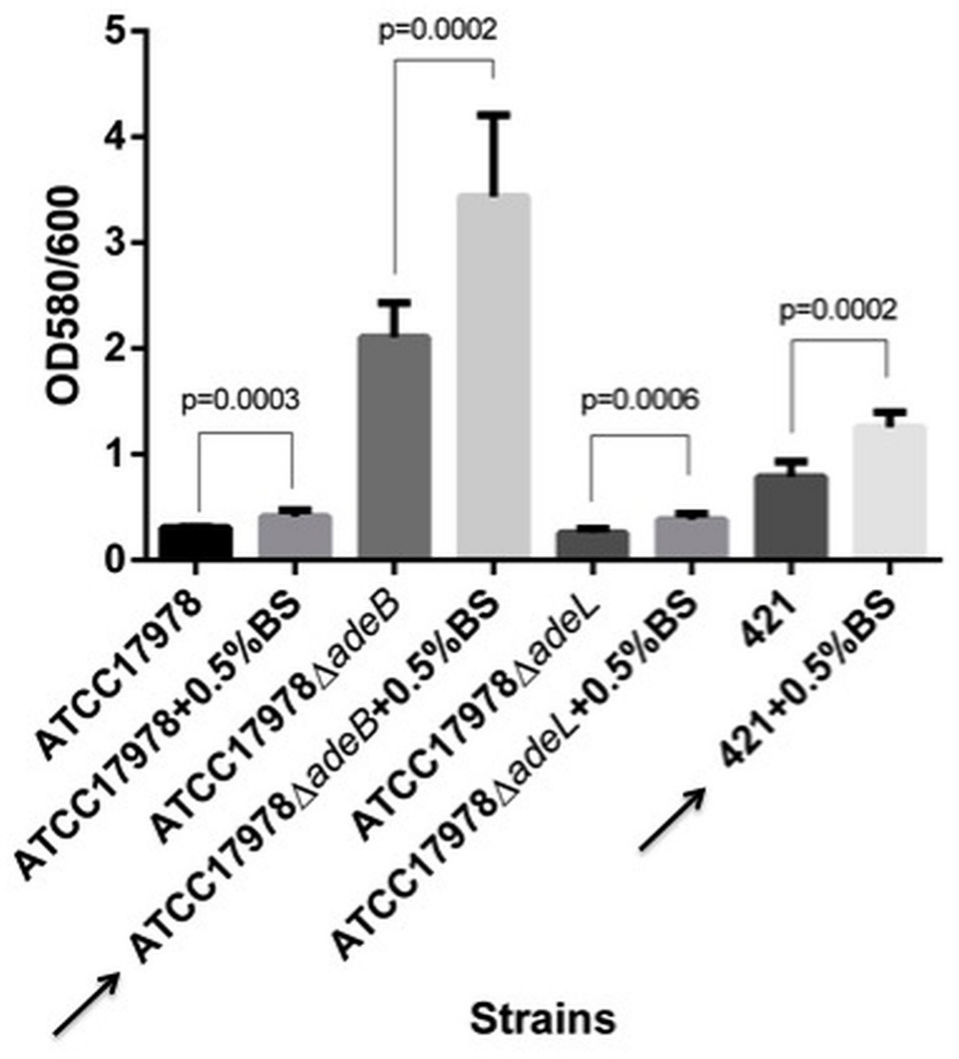
**Quantification of biofilm formation by crystal violet staining**. Eight independent replicates were considered. Results were analyzed by a Student's *t*-test. The values are means and bars indicate the standard deviation. Arrows indicate the highest biofilm producing strains cultured in the presence of bile salts (BS): *A. baumannii* Δ*adeB* ATCC 17978 and Ab421 GEIH-2010.

In the biofilm assays, the presence of bile salts (0.5% in LB-LN) increased the capacity of biofilm production in Scanning Electron Microscopy (SEM) studies (Figure 2) and quantitative assays (Student's *t*-test, *P* < 0.05) in all strains considered in this study (Figure 3). However, different phases of biofilm formation were observed in the biofilm matrix in Ab421 GEIH-2010 and microcolonies (slime layer) in *A. baumannii* Δ*adeB* ATCC 17978 cells (Figure 2: SEM analysis). Moreover, these isolates showed the highest capacity for biofilm production in quantitative assays (Figure 3).

### Gene expression in relation to bile salts in the *A. baumannii* ATCC 17978 isogenic model: microarray analysis

Microarray analysis yielded the following results (GEO database GSE85264).

A) Comparison of *A. baumannii* ATCC 17978 Isogenic Strains Cultured in the Presence of 0.5% Bile Salts (LB-LN)

Gene expression in the presence of bile salts (0.5%) in isogenic *A. baumannii* strains is summarized in Table 2. In the first experiment comparing *A. baumannii* ATCC 17978Δ*adeL* and the *A. baumannii* wild type strain, only one gene was overexpressed (*lysR* regulator family) in the mutant strain. This may be related to tolerance to bile salts in this isolate, although this was not confirmed. However, comparison of *A. baumannii* ATCC 17978Δ*adeB* and the *A. baumannii* wild type strain showed that seven genes were overexpressed in mutant strains in relation to acid tolerance (glutamate/aspartate transporter), gene mobility (transposases) and surface motility/biofilm formation (csuA/B). Finally, 25 genes associated with acid tolerance (glutamate/aspartate transporters), quorum sensing (acyl-CoA dehydrogenase, acyl-CoA synthase/AMP-acid ligases II, amino acid adenylation, acyl carrier protein, and RND superfamily transporter), iron/sulfur metabolism (ring hydroxylating dioxygenase Rieske [2Fe-2S] and aromatic-ring-hydroxylating dioxylating dioxygenase ß subunit), gene mobility (transposases), T6SS/Type VI Secretion System (vipA, hcp-1, putative signal peptide, putative membrane, and vipB), and motility/biofilm formation (csuA/B and fimbrial protein) were revealed by comparing *A. baumannii* ATCC 17978Δ*adeB* and *A. baumannii* ATCC 17978Δ*adeL*.

B) Comparison of Each Isolate Cultured in the Absence and Presence of Bile Salts (LB-LN)

**Table 2:** **Microarray analysis of the expression of genes isolated from *Acinetobacter baumanni* isogenic strains cultured in the presence of bile salts (0.5%)**.

**Gene name**	**Protein Description**	**Fold**	**Function**
***Acinetobacter baumannii*** **ATCC 17978Δ*adeL* vs. *Acinetobacter baumannii* ATCC 17978**			
A1S_2303	LysR regulator family	2.50	Regulatory
***Acinetobacter baumannii*** **ATCC 17978Δ*adeB* vs. *Acinetobacter baumannii* ATCC 17978**			
A1S_1490	Glutamate/Aspartate transporter	1.50	Acid tolerance
A1S_0658	Transposase (ISAba1)	2.57	Mobility of genes
A1S_0657	Transposase (ISAba2)	2.21	
A1S_2218	CsuA/B	1.76	Surface motility/biofilm
A1S_1071	Hypothetical protein	1.88	–
A1S_2652	Hypothetical protein	1.88	
A1S_3020	Hypothetical protein	1.84	
***Acinetobacter baumannii*** **ATCC 17978Δ*adeB* vs. *Acinetobacter baumannii* ATCC 17978Δ*adeL***			
A1S_1493	Glutamate/aspartate transport protein	1.60	Acid tolerance
A1S_1490	Glutamate/aspartate transport protein	1.59	
A1S_1492	Glutamate/aspartate transport protein	1.58	
A1S_0113	Acyl-CoA dehydrogenase	1.73	Quorum sensing
A1S_0112	Acyl-CoA synthetase/AMP-acid ligases II	1.65	
A1S_0115	Amino acid adenylation	1.52	
A1S_0114	Acyl carrier protein	1.50	
A1S_0116	RND superfamily transporter	1.50	
A1S_1860	Ring hydroxylating dioxygenase Rieske (2Fe-2S)	1.54	Iron/Sulfur metabolism
A1S_1859	Aromatic-ring-hydroxylating dioxygenase ß subunit^*a*^	1.50	
A1S_0658	Transposase (ISAba1)	2.74	Mobility of genes
A1S_0657	Transposase (ISAba2)	2.41	
A1S_1294	Type VI secretion system-associated protein (VipA)	2.05	T6SS
A1S_1296	Type VI secretion system-associated protein (Hcp-1)	1.79	
A1S_1292	Putative signal peptide	1.64	
A1S_1295	Type VI secretion system-associated protein (Putative Membrane protein)	1.62	
A1S_1293	Type VI secretion system-associated protein (VipB)	1.99	
A1S_2218	CsuA/B	1.57	Surface motility/biofilm
A1S_1510	Fimbrial protein (type I)	1.55	
A1S_1865	Glu-tRNA amidotransferase	1.64	Transferase activity
A1S_1466	Glutaminase-aspirginase	1.58	
A1S_1071	Hypothetical protein	1.92	–
A1S_2652	Hypothetical protein	1.91	
A1S_3020	Hypothetical protein	1.80	

Use of arrays to investigate gene expression in *A. baumannii* ATCC and mutant strains (*A. baumannii* Δ*adeB* ATCC 17978 and *A. baumannii* Δ*adeL* ATCC 17978) cultured in the absence and presence of bile salts (0.5%) revealed similar gene expression in all three isolates. Genes involved in the following processes were expressed under stress conditions: (i) Ferric iron binding (ii) Oxidoreductase/Transferase (iii) Isomerase/Fumarylacetoacetate, (iv) Response to toxic substances, and (v) DNA metabolism (Table 3).

**Table 3:** **Microarray analysis of the expression of genes isolated from *Acinetobacter baumanni* isogenic strains cultured in the presence (0.5%) and absence of bile salts**.

**Gene name**	**Protein Description**	**Fold**	**Function**
***Acinetobacter baumannii*** **ATCC 17978 vs**. ***Acinetobacter baumannii*** **ATCC 17978 (0.5 % Bile Salts)**			
A1S_3175	Bacterioferritin	5.21	Ferric iron binding
A1S_0800	Bacterioferritin	2.71	
A1S_1860	Ring hydroxylating dioxygenase Rieske (2Fe-2S) protein	2.27	Iron/Sulfur metabolism
A1S_1859	Aromatic-ring-hydroxylating dioxygenase beta subunit	2.21	
A1S_2102	Aldehyde dehydrogenase 1	3.57	Oxidoreductase activity
A1S_1864	Acyl-CoA dehydrogenase-like protein	2.34	
A1S_1858	Short-chain dehydrogenase/reductase SDR	2.17	
A1S_1865	Glu-tRNA amidotransferase	2.66	Transferase activity
A1S_3415	Maleylacetoacetate isomerase	2.5	Isomerase activity
A1S_1857	Vanillate O-demethylase oxidoreductase	2.31	Catalytic activity
A1S_3414	Fumarylacetoacetase	2.14	Fumarylacetoacetate activity
A1S_2809	Bacteriolytic lipoprotein entericidin B	2.03	Response to toxic substance
A1S_1228	Cold shock protein	2	DNA binding
A1S_1924	Cytochrome d terminal oxidase polypeptide subunit I	2.34	Component of membrane
***Acinetobacter baumannii*** **ATCC 17978**Δ***adeB*** **vs**. ***Acinetobacter baumannii*** **ATCC 17978**Δ***adeB*** **(0.5% Bile Salts)**			
A1S_3175	Bacterioferritin	4.17	Ferric iron binding
A1S_0800	Bacterioferritin	3.64	
A1S_1860	Ring hydroxylating dioxygenase Rieske (2Fe-2S) protein	2.79	Iron/Sulfur Metabolism
A1S_1859	Aromatic-ring-hydroxylating dioxygenase beta subunit	2.73	
A1S_1861	Benzoate dioxygenase large subunit	2.43	
A1S_2102	Aldehyde dehydrogenase 1	2.39	Oxidoreductase activity
A1S_1864	Acyl-CoA dehydrogenase-like protein	2.79	
A1S_1858	Short-chain dehydrogenase/reductase SDR	2.53	
A1S_1075	D-amino-acid dehydrogenase	2.62	
A1S_1856	P-hydroxyphenylacetate hydroxylase C1:reductase component	2.01	
A1S_1865	Glu-tRNA amidotransferase	2.97	Transferase activity
A1S_3415	Maleylacetoacetate isomerase	2.59	Isomerase activity
A1S_1857	Vanillate O-demethylase oxidoreductase	2.47	Catalytic activity
A1S_3414	Fumarylacetoacetase	2.12	Fumarylacetoacetate activity
A1S_0804	Trehalose-6-phosphate phosphatase	3.66	Metal ion binding
A1S_1498	TetR family transcriptional regulator	2.28	Transcriptional regulator
A1S_1687	Transcriptional regulator		
A1S_3416	Glyoxalase/bleomycin resistance protein/dioxygenase	2.11	Dioxygenase activity
A1S_1773	RND family drug transporter	2.04	Integral component membrane
A1S_1228	Cold shock protein	4.27	DNA binding
***Acinetobacter baumannii*****ATCC 17978**Δ***adeL*** **vs**. ***Acinetobacter baumannii*** **ATCC 17978**Δ***adeL*** **Bile Salts (0.5% Bile Salts)**			
A1S_3175	Bacterioferritin	7.63	Ferric iron binding
A1S_0800	Bacterioferritin	5.5	
A1S_2102	Aldehyde dehydrogenase 1	2.59	Oxidoreductase activity
A1S_1865	Glu-tRNA amidotransferase	2.26	Transferase activity
A1S_3415	Maleylacetoacetate isomerase	3.9	Isomerase activity
A1S_3317	Putative outer membrane protein	2.68	Component of membrane
A1S_3414	Fumarylacetoacetase	2.89	Fumarylacetoacetate activity
A1S_2809	Bacteriolytic lipoprotein entericidin B	2.76	Response to toxic substance
A1S_1687	Transcriptional regulator	2.68	Transcriptional regulator
A1S_3416	Glyoxalase/bleomycin resistance protein/dioxygenase	3.17	Dioxygenase activity
A1S_1962	Recombinase A	2.84	DNA metabolism
A1S_1224	Curved DNA-binding protein	2.16	
A1S_1228	Cold shock protein	4.92	
A1S_1031	DNA-binding ATP-dependent protease La	2.75	ATP binding
A1S_1030	DNA-binding ATP-dependent protease La	2.26	
A1S_1950	Putative universal stress protein	2.36	Response to stress
A1S_0364	Transposase	2.18	Mobility of genes
A1S_0683	Putative sigma (54) modulation protein RpoX	2.12	General metabolism
A1S_1987	Putative UDP-galactose 4-epimerase (GalE-like)	2.12	

Both results (A and B) of the microarray analysis are consistent with findings of motility studies in modified LB-LN (Figure 1).

### Growth curves on presence of bile salts (0.5% in LB-LN) in clinical strains of the ST79/PFGE-HUI-1 clone

To analyze the results obtained with isogenic models and Ab421 GEIH-2010, we used five clinical strains of *A. baumannii* belonging to the clone ST79/PFGE-HUI-1 (including the Ab421 GEIH-2010).

**Figure 4:**
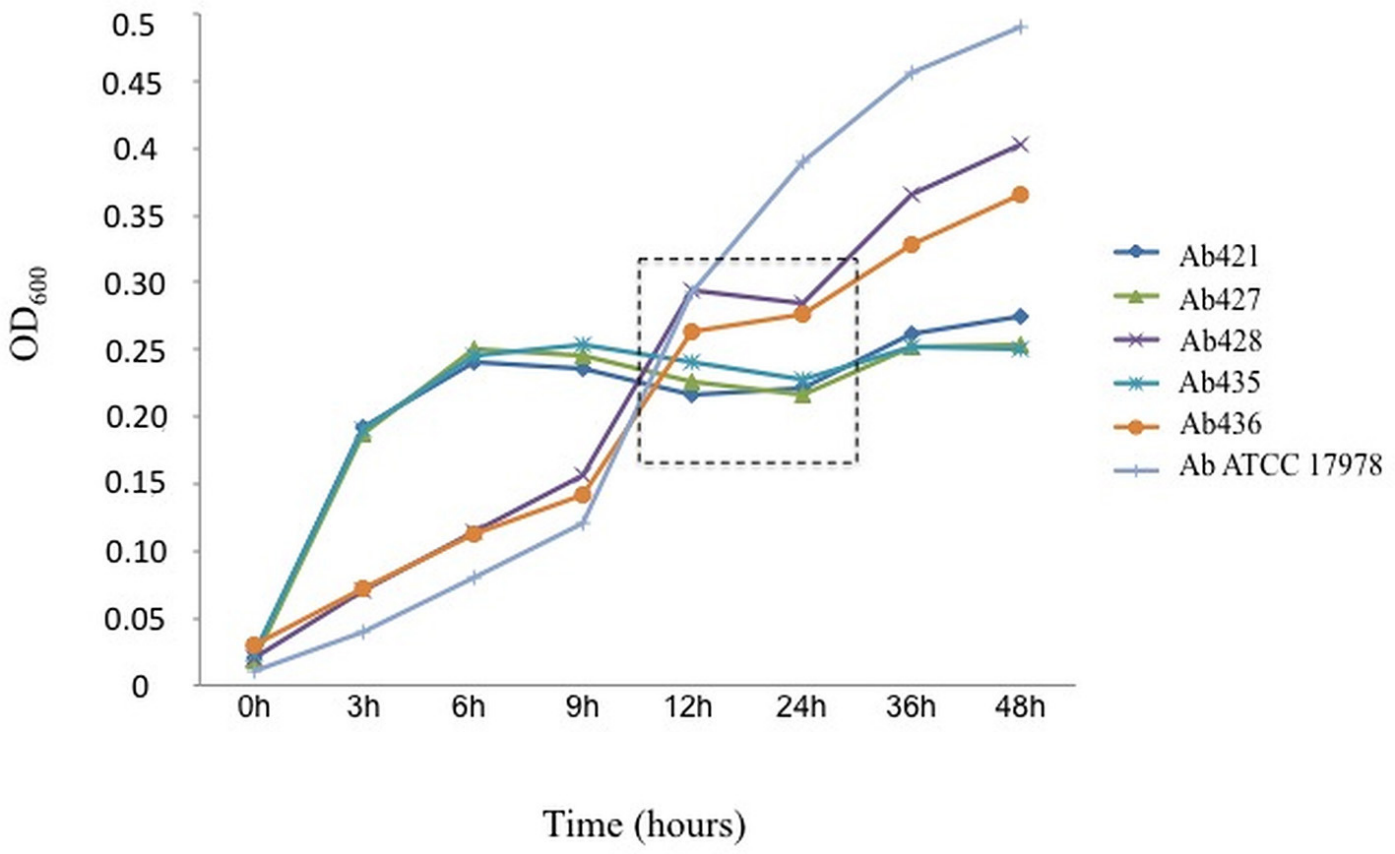
**Curves showing growth of the *A. baumannii* isolates belonging to clone ST79/PFGE-HUI-1 in the presence of bile salts (0.5%) in LB-LN**. *A. baumannii* ATCC 17978 was included as a control.

Interestingly, in the first 12 h, the *A. baumannii* clinical strains from PFGE-HUI-1 clone showed a greater growth than the strain *A. baumannii* ATCC 17978. However, after this time, the clinical strains had a stagnation (from 12 to 24 h). After 24 h, *A. baumannii* clinical strains from PFGE-HUI-1 and the *A. baumannii* ATCC 17978 strain reached to an OD_600_ of 0.5 and 0.3 (the bacterial growth was inoculated on LB plates). This may indicate the development of biofilm formation in the clinical strains from PFGE-HUI-1 clone not being possible the study of growth by optical density (Figure 4).

### Gene expression in relation to bile salts in clinical strains of ST79/PFGE-HUI-1 clone as revealed by RT-PCR

Gene overexpression detected by microarray analysis was confirmed by RT-PCR in clinical strains of clone ST79/PFGE-HUI-1 (in addition to the Ab421 GEIH-2010). The levels of overexpression (RE) of A1S_1490 (Acid tolerance), A1S_0115 (Quorum Sensing), A1S_1295 (T6SS), and A1S_1510 (Surface motility/Biofilm formation) were statistically significantly higher in clinical isolates of the ST79/PFGE-HUI-1 clone cultured in the presence of bile salts (0.5%) than in the same clone cultured in LB-LN (Table 4). These results obtained with the clinical strains of the PFGE-HUI clone confirmed the overexpression of genes involved in the response to bile salts in the isogenic model of *A. baumannii* ATCC 17978. Moreover, all strains of this ST79/PFGE-HUI-1 clone (17 isolates) were considered to be the cause of infection in all six patients. Interestingly, five of the six patients had bacteraemia (primary or secondary).

**Table 4:** **Expression of genes involved in Acid tolerance, Quorum sensing, T6SS, and surface motility/biofilm mechanisms in clinical strains (clone ST79/PFGE-HUI) cultured in the presence of bile salts (0.5%), determined by RT-PCR**.

***A.baumannii*** **GEIH-2010 (PFGE-HUI-1 clone)**	**Acid tolerance (*A1S_1490* gene^a^)**	**Quorum sensing (*A1S_0115* gene^a^)**	**T6SS (*A1S_1295* gene^a^)**	**Surface motility/biofilm (*A1S_1510* gene^a^)**	**Bacteraemia**
Ab421	4.16	4.28	3.38	3.83	Yes
Ab427	2.42	1.68	2.28	2.42	Yes
Ab428	2.60	1.87	2.36	2.41	Yes
Ab435	9.00	9.78	7.83	8.39	Yes
Ab436	4.72	2.80	3.27	4.31	Yes
Control^*^	**Acid tolerance (*****A1S_1490*** **gene**^a^**)**	**Quorum sensing (*****A1S_0115*** **gene**^a^**)**	**T6SS (*****A1S_1295*** **gene**^a^**)**	**Surface motility/biofilm (*****A1S_1510*** **gene**^a^**)**	
*A. baumannii* ATCC 17978	1.23	1.03	1.10	1.05	–

* *A. baumannii ATCC 17978 was included as a control. The same grown in the absence of bile salts were used as references strains (RE = 1)*.

a *Genome A. baumannii ATCC 17978*.

## Discussion

The RND type of multidrug efflux pumps play several roles in bacterial pathogens: (i) provision of resistance to antimicrobial and antiseptic compounds, including those naturally present in mucosa; (ii) regulation of virulence factors via involvement in quorum-sensing regulation; (iii) detoxification of intracellular metabolites; and finally, (iv) mediation of cell homeostasis and intercellular signal trafficking (Beceiro et al., 2013).

In *A. baumannii*, the AdeABC RND-pump is the main efflux system involved in antimicrobial resistance. However, 25–30% of clinical strains of *A. baumannii* do not possess the AdeABC efflux pump (Chu et al., 2006; Lin et al., 2009).

In the GEIH-REIPI-2010 Ab Project (a multicentre study in which 45 hospitals participated), we studied clinical strain Ab421 GEIH-2010, which lacks the AdeABC efflux pump as well as regulatory genes and also overexpresses the AdeFGH system. This clinical strain belongs to clone ST79/PFGE-HUI-1 (seventeen isolates) (Rumbo et al., 2013). The mechanisms associated to the adaptive response to bile salts were analyzed in mutant strains of *A. baumannii* ATCC 17978 by considering an isogenic model (*A. baumannii* Δ*adeB* ATCC 17978 and *A. baumannii* Δ*adeL* ATCC 17978). Interestingly, the Ab421 GEIH-2010 strain displayed a higher capacity for surface motility and biofilm formation when cultured in the presence of bile salts than when cultured in the absence of these.

The mechanisms associated to the response to bile salts (essential for survival in the gastrointestinal tract) are not well-known in *A. baumannii*. In a study of the profile of protein overexpression in response to monovalent cations (200 mM of NaCl), Hood et al. observed glutamate/aspartate transport (Hood et al., 2010). Moreover, glutamate transport (glnQHMP operon) has also been implicated with acid tolerance in *Streptococcus mutans* (Krastel et al., 2010). Our results with *A. baumannii* Δ*adeB* ATCC 17978 (microarray analysis) and clinical strains of ST79/PFGE-HUI-1, including strain Ab421 GEIH-2010 (RT-PCR), revealed the involvement of this transporter and associated proteins in tolerance to physiological concentrations of bile salts (Prouty et al., 2004; Sánchez et al., 2005).

Virulence factors associated with activation of the QS system may be regulated differently throughout the intestine depending on the bile salts present, the level of tolerance and the presence of different commensal bacteria (Bachmann et al., 2015). We observed overexpression of the QS genes (A1S_0112 to A1S_0116) in strains lacking the AdeABC efflux pump (*A. baumannii* Δ*adeB* ATCC 17978 and clinical strains of ST79/PFGE-HUI-1) cultured in the presence of bile salts. In a study using transcriptomic analysis, Clemmer et al. confirmed that this cluster of genes was induced by the 3-OH C12-HSL molecule (a signal in the QS network). Interestingly, the A1S_0116 gene encodes a RND superfamily transporter which may be involved in efflux of the molecules from the QS system (Clemmer et al., 2011). Moreover, virulence factors modulated by the QS system were overexpressed, i.e., surface motility, biofilm formation (A1S_2218 and A1S_1510) (Clemmer et al., 2011; Rumbo-Feal et al., 2013) and the Type VI Secretion System (T6SS) (A1S_1292 to A1S_1296) (Sana et al., 2012).

Zheng et al., investigated the role of the QS system in *Vibrio cholerae* in modulating the expression of virulence factors such as T6SS (Zheng et al., 2010). The authors established that a high density of bacteria is critical for expression of the T6SS pandemic *V. cholerae* C6706. The T6SS may be a potent mediator of survival of the pathogen or commensals in multi-bacterial environments, biofilms and in polymicrobial infections, such as those encountered in the airways of cystic fibrosis patients (Rumbo-Feal et al., 2013; Bachmann et al., 2015) and in gastrointestinal colonization in these patients (Bachmann et al., 2015). Three T6SS systems (H1, H2, and H3-T6SS) with functions at different stages of the infection process (colonization vs. dissemination) or the infection mode (acute virulence vs. chronic persistence) have been described in *Pseudomonas aeruginosa* strain PAO1 (Jani and Cotter, 2010; Schwarz et al., 2010). Moreover, H2-T6SS is regulated by QS in this pathogen (Sana et al., 2012).

Several studies have investigated the role of the T6SS system in *A. baumannii* strains. Repizo et al., reported the first case of *A. baumannii* environmental strain DSM30011 in which the T6SS system was implicated in host colonization (Repizo et al., 2015). Recently, Weber and collaborators demonstrated expression of the T6SS system in *A. baumannii* clinical strains susceptible to several antibiotics (Weber et al., 2015). The authors explained that T6SS is an antibacterial system used by Gram-negative bacteria to kill competitors. The *A. baumanni* clinical strains carry T6SS repressors (TetR regulators) in plasmids harboring resistance genes that prevent expression of these T6SS systems (Weber et al., 2015, 2016). When Multi-Drug-Resistant (MDR) strains of *A. baumannii* are not exposed to antibiotics, such as in the inanimate hospital environment or in untreated polymicrobial infections, there is an increased likelihood of encountering competitors that will activate bacterial T6SS systems (Weber et al., 2015). In Spanish hospitals, ST79/PFGE-HUI-1 was the only carbapenem-susceptible clone that did not possess a plasmid carrying OXA 24 ß-lactamase or the AbKAB Toxin-Antitoxin system (Rumbo et al., 2013; Mosqueda et al., 2014). In the present study, resistance to antimicrobials (aminoglycosides, quinolones and glycines) in Ab421 GEIH-2010 (belonging to ST79/PFGE-HUI-1) and *A. baumannii* Δ*adeL* ATCC 17978 was associated with overexpression of the AdeFGH pump.

Finally, although only a small number of cases were considered (out to seven), all patients from whom strains of *A. baumannii* clone ST79/PFGE-HUI-1 were isolated had infections and five out of patients developed bacteraemia (72%). This contrasts with usual observations for *A. baumannii*, as about half of colonized patients do not usually have infections due to the pathogen (Cisneros and Rodríguez-Baño, 2002; Villar et al., 2014), and the prevalence of bacteraemia in infected patients is usually lower than 10% (Thom et al., 2010). Thom and collaborators found that in 86% of patients from ICUs who had the gastrointestinal tract colonized by *A. baumannii* clinical strains, they had bacteraemia through genetically similar strains (Thom et al., 2010). This implies that those clinical isolates of *A. baumannii* that present a higher capacity to survive gastrointestinal conditions (including bile salts tolerance) through biofilm formation and others mechanisms as transporters could present an increase in their invasive capacity (development of bacteraemia) due to virulence factors (as the type VI secretion system) previously activated under pressure conditions.

In conclusion, this is the first study about the adaptive response to bile salts investigating the molecular and microbiological characteristics in response to bile salts of an isogenic model of *A. baumannii* ATCC 17978 (*A. baumannii* Δ*adeB* ATCC 17978) and clinical isolates of *A. baumannii* (clinical strains of ST79/PFGE-HUI-1) lacking the main RND efflux pump (AdeABC). The response to bile salts led to activation of the QS system and modulated virulence factors such as surface motility, biofilm and Type VI secretion system. Moreover, we observed a new clinical profile (increased invasiveness) of strains of *A. baumannii* ST79/PFGE-HUI-1 lacking the AdeABC efflux pump. Further studies should be carried out with clinical strains of *A. baumannii* with or without the AdeABC efflux pump (or in which the pump is inhibited by treatment) (Pannek et al., 2006; Blair and Piddock, 2009; López et al., 2014; Richmond et al., 2016) under others stress conditions (oxidative and osmotic) that may activate global mechanisms such as the QS system, which modulates virulence factors.

## Nucleotide sequence accession number

The genome sequence of Ab421 GEIH-2010 strain has been deposited at GenBank under accession number CP014266.1. This genome sequence was determined as part of a II Spanish multicenter study, GEIH-REIPI *A. baumannii* 2000–2010 project (PRJNA308422).

## Author contributions

Funding acquisition: MT; Investigation: ML, LB, EG, LF, LM, FF, JR, AP (4th author), AP (9th author), GB, and MT; Methodology: ML, LB, EG, LF; Supervision: MT; Writing: MT.

## Funding

This study was funded by grant PI13/02390 and PI16/01163 awarded to MT within the State Plan for R+D+I 2013-2016 (National Plan for Scientific Research, Technological Development and Innovation 2008-2011) and co-financed by the ISCIII-Deputy General Directorate of evaluation and Promotion of Research—European Regional Development Fund “A way of Making Europe” and Instituto de Salud Carlos III FEDER, Spanish Network for Research in Infectious Diseases (REIPI RD12/0015). MT was financially supported by the Miguel Servet Research Programme (C.H.U.A Coruña and ISCIII).

### Conflict of interest statement

The authors declare that the research was conducted in the absence of any commercial or financial relationships that could be construed as a potential conflict of interest.

## Supplementary material

Table S1 **Primers and probes used in this study (Supplementary material)**.

## References

[B1] Álvarez-Fraga L. Pérez A. Rumbo-Feal S. Merino M. Vallejo J. A. Ohneck E. J.. (2016). Analysis of the role of the LH92_11085 gene of a biofilm hyper-producing *Acinetobacter baumannii* strain on biofilm formation and attachment to eukaryotic cells Virulence 7, 443–455. 10.1080/21505594.2016.1145335 26854744 PMC4871663

[B2] Antunes L. C. Visca P. Towner K. J. (2013). *Acinetobacter baumannii*: evolution of a global pathogen. Pathog. Dis. 71, 292–301. 10.1111/2049-632X.12125 24376225

[B3] Aranda J. Poza M. Shingu-Vázquez M. Cortés P. Boyce J. D. Adler B.. (2013). Identification of a DNA-damage-inducible regulon in *Acinetobacter baumannii*. J. Bacteriol. 195, 5577–5582. 10.1128/JB.00853-13 24123815 PMC3889614

[B4] Bachmann V. Kostiuk B. Unterweger D. Diaz-Satizabal L. Ogg S. Pukatzki S. (2015). Bile salts modulate the mucin-activated type VI secretion system of pandemic *Vibrio cholerae*. PLoS Negl. Trop. Dis. 9:e0004031. 10.1371/journal.pntd.0004031 26317760 PMC4552747

[B5] Beceiro A. Tomás M. Bou G. (2013). Antimicrobial resistance and virulence: a successful or deleterious association in the bacterial world? Clin. Microbiol. Rev. 26, 185–230. 10.1128/CMR.00059-12 23554414 PMC3623377

[B6] Blair J. M. Piddock L. J. (2009). Structure, function and inhibition of RND efflux pumpsin gram-negativebacteria: an update. Curr. Opin. Microbiol. 12, 512–519. 10.1016/j.mib.2009.07.003 19664953

[B7] Cefai C. Richards J. Gould F. K. McPeake P. (1990). An outbreak of *Acinetobacter* respiratory tract infection resulting from incomplete disinfection of ventilatory equipment. J. Hosp. Infect. 15, 177–182. 10.1016/0195-6701(90)90128-B 1969441

[B8] Chu Y. W. Chau S. L. Houang E. T. (2006). Presence of active efflux systems AdeABC, AdeDE and AdeXYZ in different *Acinetobacter* genomic DNA groups. J. Med. Microbiol. 55(Pt 4), 477–478. 10.1099/jmm.0.46433-0 16534000

[B9] Cisneros J. M. Rodríguez-Baño J. (2002). Nosocomial bacteremia due to *Acinetobacter baumannii*: epidemiology, clinical features and treatment. Clin. Microbiol. Infect. 8, 687–693. 10.1046/j.1469-0691.2002.00487.x 12445005

[B10] Clemmer K. M. Bonomo R. A. Rather P. N. (2011). Genetic analysis of surface motility in *Acinetobacter baumannii*. Microbiology 157(Pt 9), 2534–2544. 10.1099/mic.0.049791-0 21700662 PMC3352170

[B11] CLSI Clinical and Laboratory Standards Institute (2015). Performance Standards for Antimicrobial Susceptibility Testing; Twenty-Fifth Informational Supplement (M100-S25).

[B12] Corbella X. Pujol M. Ayats J. Sendra M. Ardanuy C. Domínguez M. A.. (1996). Relevance of digestive tract colonization in the epidemiology of nosocomial infections due to multiresistant *Acinetobacter baumannii*. Clin. Infect. Dis. 23, 329–334. 10.1093/clinids/23.2.329 8842272

[B13] Coyne S. Courvalin P. Perichon B. (2011). Efflux-mediated antibiotic resistance in *Acinetobacter* spp. Antimicrob. Agents Chemother. 55, 947–953. 10.1128/AAC.01388-10 21173183 PMC3067115

[B14] Coyne S. Rosenfeld N. Lambert T. Courvalin P. Perichon B. (2010). Overexpression of resistance-nodulation-cell division pump AdeFGH confers multidrug resistance in *Acinetobacter baumannii*. Antimicrob. Agents Chemother. 54, 4389–4393. 10.1128/AAC.00155-10 20696879 PMC2944555

[B15] del Mar Tomas M. Cartelle M. Pertega S. Beceiro A. Llinares P. Canle D.. (2005). Hospital outbreak caused by a carbapenem-resistant strain of *Acinetobacter baumannii*: patient prognosis and risk-factors for colonisation and infection. Clin. Microbiol. Infect. 11, 540–546. 10.1111/j.1469-0691.2005.01184.x 15966971

[B16] Gaddy J. A. Tomaras A. P. Actis L. A. (2009). The *Acinetobacter baumannii* 19606 OmpA protein plays a role in biofilm formation on abiotic surfaces and in the interaction of this pathogen with eukaryotic cells. Infect. Immun. 77, 3150–3160. 10.1128/IAI.00096-09 19470746 PMC2715673

[B17] Hamad M. A. Zajdowicz S. L. Holmes R. K. Voskuil M. I. (2009). An allelic exchange system for compliant genetic manipulation of the select agents *Burkholderia pseudomallei* and *Burkholderia mallei*. Gene 430, 123–131. 10.1016/j.gene.2008.10.011 19010402 PMC2646673

[B18] Hamner S. McInnerney K. Williamson K. Franklin M. J. Ford T. E. (2013). Bile salts affect expression of *Escherichia coli* O157:H7 genes for virulence and iron acquisition, and promote growth under iron limiting conditions. PLoS ONE 10:e74647 10.1371/journal.pone.0074647 PMC3769235 24058617

[B19] He X. Lu F. Yuan F. Jiang D. Zhao P. Zhu J.. (2015). Biofilm formation caused by clinical *Acinetobacter baumannii* isolates is associated with overexpression of the AdeFGH Efflux pump. Antimicrob. Agents Chemother. 59, 4817–4825. 10.1128/AAC.00877-15 26033730 PMC4505227

[B20] Hood M. I. Jacobs A. C. Sayood K. Dunman P. M. Skaar E. P. (2010). *Acinetobacter baumannii* increases tolerance to antibiotics in response to monovalent cations. Antimicrob. Agents Chemother. 54, 1029–1041. 10.1128/AAC.00963-09 20028819 PMC2825970

[B21] Jani A. J. Cotter P. A. (2010). Type VI secretion: not just for pathogenesis anymore. Cell Host Microbe 8, 2–6. 10.1016/j.chom.2010.06.012 20638635 PMC2913581

[B22] Jin L. Z. Ho Y. W. Abdullah N. Jalaludin S. (1998). Acid and bile tolerance of *Lactobacillus* isolated from chicken intestine. Lett. Appl. Microbiol. 27, 183–185. 10.1046/j.1472-765X 9750324

[B23] Krastel K. Senadheera D. B. Mair R. Downey J. S. Goodman S. D. Cvitkovitch D. G. (2010). Characterization of a glutamate transporter operon, glnQHMP, in *Streptococcus mutans* and its role in acid tolerance. J. Bacteriol. 192, 984–993. 10.1128/JB.01169-09 20023025 PMC2812961

[B24] Lin J. Sahin O. Michel L. O. Zhang Q. (2003). Critical role of multidrug efflux pump CmeABC in bile resistance and *in vivo* colonization of *Campylobacter jejuni*. Infect. Immun. 71, 4250–4259. 10.1128/IAI.71.8. 4250-4259.2003. 12874300 PMC165992

[B25] Lin L. Ling B. D. Li X. Z. (2009). Distribution of the multidrug efflux pump genes, adeABC, adeDE and adeIJK, and class 1 integron genes in multiple-antimicrobial-resistant clinical isolates of *Acinetobacter baumannii*-*Acinetobacter calcoaceticus* complex. Int. J. Antimicrob. Agents 33, 27–32. 10.1016/j.ijantimicag.2008.06.027 18790612

[B26] Lopez M. Álvarez-Fraga L. Gato E. Blasco L. Poza M. Fernández-García L.. (2016). Genome sequence of a clinical strain of *Acinetobacter baumannii* belonging to the ST79/PFGE-HUI-1 clone lacking the AdeABC (resistance-nodulation-cell division-type) efflux pump. Genome Announc. 4:e00962-16. 10.1128/genomeA.00962-16 27609928 PMC5017233

[B27] López M. Barbosa B. Gato E. Bou G. Tomas M. (2014). Patents on antivirulence therapies. World J. Pharmacol. 3, 97–109. 10.5497/WJP.v3.i4.97

[B28] López M. Mayer C. Fernández-García L. Blasco L. Muras A. Ruiz F. M.. (2017). Quorum sensing network in clinical strains of *A. baumannii*: AidA is a new quorum quenching enzyme. PLoS ONE 12:e0174454. 10.1371/journal.pone.0174454 28328989 PMC5362224

[B29] Malik N. A. (2016). Solubilization and interaction studies of bile salts with surfactants and drugs: a review. Appl. Biochem. Biotechnol. 179, 179–201. 10.1007/s12010-016-1987-x 26781714

[B30] Mosqueda N. Gato E. Roca I. López M. de Alegría C. R. Fernández Cuenca F.. (2014). Characterization of plasmids carrying the blaOXA-24/40 carbapenemase gene and the genes encoding the AbkA/AbkB proteins of a toxin/antitoxin system. J. Antimicrob. Chemother. 69, 2629–2633. 10.1093/jac/dku179 24879663

[B31] Mulin B. Talon D. Viel J. F. Vincent C. Leprat R. Thouverez M.. (1995). Risk factors for nosocomial colonization with multiresistant *Acinetobacter baumannii*. Eur. J. Clin. Microbiol. Infect. Dis. 14, 569–576. 10.1007/BF01690727 7588840

[B32] Pannek S. Higgins P. G. Steinke P. Jonas D. Akova M. Bohnert J. A.. (2006). Multidrug efflux inhibition in *Acinetobacter baumannii*: comparison between 1-(1-naphthylmethyl)-piperazine and phenyl-arginine-beta-naphthylamide. J. Antimicrob. Chemother. 57, 970–974. 10.1093/jac/dkl081 16531429

[B33] Prouty A. M. Brodsky I. E. Falkow S. Gunn J. S. (2004). Bile-salt-mediated induction of antimicrobial and bile resistance in *Salmonella typhimurium*. Microbiology 150(Pt 4), 775–783. 10.1099/mic.0.26769-0 15073288

[B34] Pumbwe L. Skilbeck C. A. Wexler H. M. (2008). Presence of quorum-sensing systems associated with multidrug resistance and biofilm formation in *Bacteroides fragilis*. Microb. Ecol. 56, 412–419. 10.1007/s00248-007-9358-3 18188535

[B35] Pumbwe L. Skilbeck C. A. Nakano V. Avila-Campos M. J. Piazza R. M. Wexler H. M. (2007). Bile salts enhance bacterial co-aggregation, bacterial-intestinal epithelial cell adhesion, biofilm formation and antimicrobial resistance of *Bacteroides fragilis*. Microb. Pathog. 43, 78–87. 10.1016/j.micpath.2007.04.002 17524609

[B36] Repizo G. D. Gagné S. Foucault-Grunenwald M. L. Borges V. Charpentier X. Limansky A. S.. (2015). Differential role of the T6SS in *Acinetobacter baumannii* virulence. PLoS ONE 10:e0138265. 10.1371/journal.pone.0138265 26401654 PMC4581634

[B37] Rey I. Soubigou P. Debussche L. David C. Morgat A. Bost P. E.. (1989). Antibodies to synthetic peptide from the residue 33 to 42 domain of c-Ha-ras p21 block reconstitution of the protein with different effectors. Mol. Cell. Biol. 9, 3904–3910. 10.1128/MCB.9.9.3904 2550807 PMC362452

[B38] Richmond G. E. Evans L. P. Anderson M. J. Wand M. E. Bonney L. C. Ivens A.. (2016). The *Acinetobacter baumannii* two-component system AdeRS regulates genes required for multidrug efflux, biofilm formation, and virulence in a strain-specific manner. MBio 7:e00852-16. 10.1128/mBio.00852-16 27094331 PMC4850262

[B39] Rodríguez-Baño J. Cisneros J. M. Fernández-Cuenca F. Ribera A. Vila J. Pascual A.. (2004). Clinical features and epidemiology of *Acinetobacter baumannii* colonization and infection in Spanish hospitals. Infect. Control Hosp. Epidemiol. 25, 819–824. 10.1086/502302 15518022

[B40] Rumbo C. Gato E. López M. Ruiz de Alegría C. Fernández-Cuenca F. Martínez-Martínez L.. (2013). Contribution of efflux pumps, porins, and β-lactamases to multidrug resistance in clinical isolates of *Acinetobacter baumannii*. Antimicrob. Agents Chemother. 57, 5247–5257. 10.1128/AAC.00730-13 23939894 PMC3811325

[B41] Rumbo-Feal S. Gómez M. J. Gayoso C. Álvarez-Fraga L. Cabral M. P. Aransay A. M.. (2013). Whole transcriptome analysis of *Acinetobacter baumannii* assessed by RNA-sequencing reveals different mRNA expression profiles in biofilm compared to planktonic cells. PLoS ONE 8:e72968. 10.1371/journal.pone.0072968 24023660 PMC3758355

[B42] Sana T. G. Hachani A. Bucior I. Soscia C. Garvis S. Termine E.. (2012). The second type VI secretion system of *Pseudomonas aeruginosa* strain PAO1 is regulated by quorum sensing and Fur and modulates internalization in epithelial cells. J. Biol. Chem. 287, 27095–27105. 10.1074/jbc.M112.376368 22665491 PMC3411052

[B43] Sánchez B. Champomier-Vergès M. C. Anglade P. Baraige F. de Los Reyes-Gavilán C. G. Margolles A.. (2005). Proteomic analysis of global changes in protein expression during bile salt exposure of *Bifidobacterium longum* NCIMB 8809. J. Bacteriol. 187, 5799–5808. 10.1128/JB.187.16.5799-5808.2005 16077128 PMC1196055

[B44] Schwarz S. Hood R. D. Mougous J. D. (2010). What is type VI secretion doing in all those bugs? Trends Microbiol. 18, 531–537. 10.1016/j.tim.2010.09.001 20961764 PMC2991376

[B45] Sherertz R. J. Sullivan M. L. (1985). An outbreak of infections with *Acinetobacter calcoaceticus* in burn patients: contamination of patients' mattresses. J. Infect. Dis. 151, 252–258. 10.1007/BF01643394 3968451

[B46] Thom K. A. Hsiao W. W. Harris A. D. Stine O. C. Rasko D. A. Johnson J. K. (2010). Patients with *Acinetobacter baumannii* bloodstream infections are colonized in the gastrointestinal tract with identical strains. Am. J. Infect. Control 38, 751–753. 10.1016/j.ajic.2010.03.005 20570393 PMC3010856

[B47] Tomaras A. P. Dorsey C. W. Edelmann R. E. Actis L. A. (2003). Attachment to and biofilm formation on abiotic surfaces by *Acinetobacter baumannii*: involvement of a novel chaperone-usher pili assembly system. Microbiology 149(Pt 12), 3473–3484. 10.1099/mic.0.26541-0 14663080

[B48] Tomas M. Doumith M. Warner M. Turton J. F. Beceiro A. Bou G.. (2010). Efflux pumps, OprD porin, AmpC beta-lactamase, and multiresistance in *Pseudomonas aeruginosa* isolates from cystic fibrosis patients. Antimicrob. Agents Chemother. 54, 2219–2224. 10.1128/AAC.00816-09 20194693 PMC2863613

[B49] Villar M. Gato E. Garnacho-Montero J. Cisneros J. M. Ruíz de Alegría C. Fernández-Cuenca F.. (2014). Epidemiological and clinical impact of Acinetobacter baumannii colonization and infection: a reappraisal. Medicine 93, 202–210. 10.1099/mic.0.26541-0 25181313 PMC4602455

[B50] Weber B. S. Hennon S. W. Wright M. S. Scott N. E. de Berardinis V. Foster L. J.. (2016). Genetic dissection of the type VI secretion system in *Acinetobacter* and identification of a novel peptidoglycan hydrolase, TagX, required for its biogenesis. MBio 7:e01253-16. 10.1128/mBio.01253-16 27729508 PMC5061870

[B51] Weber B. S. Ly P. M. Irwin J. N. Pukatzki S. Feldman M. F. (2015). A multidrug resistance plasmid contains the molecular switch for type VI secretion in *Acinetobacter baumannii*. Proc. Natl. Acad. Sci. U.S.A. 112, 9442–9447. 10.1073/pnas.1502966112 26170289 PMC4522760

[B52] Yoon E. J. Chabane Y. N. Goussard S. Snesrud E. Courvalin P. Dé E.. (2015). Contribution of resistance-nodulation-cell division efflux systems to antibiotic resistance and biofilm formation in *Acinetobacter baumannii*. MBio 6:e00309-15. 10.1128/mBio.00309-15 25805730 PMC4453527

[B53] Zheng J. Shin O. S. Cameron D. E. Mekalanos J. J. (2010). Quorum sensing and a global regulator TsrA control expression of type VI secretion and virulence in *Vibrio cholerae*. Proc. Natl. Acad. Sci. U.S.A. 107, 21128–21133. 10.1073/pnas.1014998107 21084635 PMC3000250

