# Supplementary material for: Response to Bile Salts in Clinical Strains of Acinetobacter baumannii Lacking the AdeABC Efflux Pump: Virulence Associated with Quorum Sensing
Source: Front Cell Infect Microbiol. 2017 May 9;7:143. doi: 10.3389/fcimb.2017.00143 (PMC5423435; doi:10.3389/fcimb.2017.00143)
Supplement: Table S1 — Primers and probes used in this study (Supplementary material). [file Table1.pdf]

MUTANTS from *A.baumannii* ATCC17978

Primer name	Sequence (5’-3’)	Strain
AdeLUp(BamHI)FW<br>AdeLUp(KpnI)RV<br>AdeLdown(KpnI)FW<br>AdeLdown(SphI)RV<br>Int-UpAdeL-FW<br>Int-DownAdeL-RV	CGCGGATCCGCGCTTTCAAGTCTGCATCTTC<br>CGGGGTACCCCGGATAAAGAAACTGGTCATGA<br>GGGGGTACCCCCGGCATGAAATAGATCCACAC<br>GGGGCATGCCCCTTGATTGTACCTTCGCGAGT<br>GGTTGGCGTATTGTAGATTT<br>GGGAAAATGACATGAGGTGC	*A. baumannii* ATCC 17978Δ*adeL*
AdeBup(NotI)FW<br>AdeBup(BglII)RV<br>AdeBdown(BglII)FW<br>AdeBdown(BamHI)RV<br>Int-UpAdeB-FW<br>Int-DownAdeB-RV	CCCGCGGCCGCGGGCCGTCCGCAAGTCGGAGG<br>CCCAGATCTGGGGGTTGCGCCCCCTCAGTT<br>GGGAGATCTGGGCGCCAAAGGGCCAATATC<br>GGGGGATCCGGG TACAGGAGTCGCTGGTTC<br>AGCGGAAATTCGTCCTATCG<br>GAGAAAGGCAACACAGTC	*A. baumannii* ATCC 17978Δ*adeB*
pMo130site2F<br>pMo130site2R	ATTCATGACCGTGCTGAC<br>CTTGTCTGTAAGCGGATG	Plasmid pMo130

RT-PCR Ab421 GEIH-2010

Primer name	Sequence (5’-3’)	UPL Probes (number/sequence)	Genes	System
A1S_1490F<br>A1S_1490R	CTTCTCAAATTCAGGCAGCA<br>ATTCTCGATGGCCAATAACG	48/ACTGGGAA	A1S_1490	Acid tolerance
A1S_0115F<br>A1S_0115R	CCGCTATGGCTGTTCCAT<br>CCGCGAAAGCTCCATATAAC	87/GGTGGCAG	A1S_0115	Quorum sensing
A1S_1295F<br>A1S_1295R	TGGTTGGTGTGCTGCTATTC<br>ATGTACTGGCAAGCCTTCAAC	77/GGTGGTGG	A1S_1295	T6SS
A1S_1510F<br>A1S_1510R	AGCATAATAATTAAGATCGGCATTC<br>GGTGCACAAGATAACTCACAATG	3/CCCAGCAG	A1S_1510	Surface motility/Biofilm
AdeGF<br>AdeGR	GTCCTGAAATGGTCGTTCGT<br>AGCTTCTGCTTGGCTAGATGA	43/CTGCCCCA	*adeG*	AdeFGH<br>(RND efflux pump)
RpoBF<br>*RpoBR*	CGTGTATCTGCGCTTGG<br>CGTACTTCGAAGCCTGCAC	131/CTGGTGGT	*rpoB*	Housekeeping

Table S1.
